# The effect of obstructive sleep apnea on peripheral blood amino acid and biogenic amine metabolome at multiple time points overnight

**DOI:** 10.1038/s41598-021-88409-y

**Published:** 2021-05-24

**Authors:** Ott Kiens, Egon Taalberg, Viktoria Ivanova, Ketlin Veeväli, Triin Laurits, Ragne Tamm, Aigar Ottas, Kalle Kilk, Ursel Soomets, Alan Altraja

**Affiliations:** 1 Department of Pulmonary Medicine, University of Tartu, Tartu, Estonia; 2 Lung Clinic, Tartu University Hospital, Tartu University Lung Clinic, Riia 167, 50411 Tartu, Estonia; 3 Institute of Biomedicine and Translational Medicine, University of Tartu, Tartu, Estonia; 4 Centre of Excellence for Genomics and Translational Medicine, University of Tartu, Tartu, Estonia; 5 Psychiatry Clinic, Tartu University Hospital, Tartu, Estonia

**Keywords:** Metabolomics, Respiratory tract diseases

## Abstract

There are no clinical studies that have investigated the differences in blood serum metabolome between obstructive sleep apnea (OSA) patients and controls. In a single-center prospective observational study, we compared metabolomic profiles in the serum of OSA patients with apnea–hypopnea index (AHI) ≥ 15/h and control individuals. Peripheral blood was obtained at 3 different time points overnight: 9:00 p.m.; 5:00 a.m. and 7:00 a.m. We used a targeted approach for detecting amino acids and biogenic amines and analyzed the data with ranked general linear model for repeated measures. We recruited 31 patients with moderate-to-severe OSA and 32 controls. Significant elevations in median concentrations of alanine, proline and kynurenine in OSA patients compared to controls were detected. Significant changes in the overnight dynamics of serum concentrations occurred in OSA: glutamine, serine, threonine, tryptophan, kynurenine and glycine levels increased, whereas a fall occurred in the same biomarker levels in controls. Phenylalanine and proline levels decreased slightly, compared to a steeper fall in controls. The study indicates that serum profiles of amino acid and biogenic amines are significantly altered in patients with OSA referring to vast pathophysiologic shifts reflected in the systemic metabolism.

## Introduction

Obstructive sleep apnea (OSA) is characterized by a history of habitual snoring and the occurrence of obstructed breathing events, hypopneas, and apneas, during sleep. OSA is a highly prevalent sleep disorder, with an estimated prevalence of 10–49.7% among adult men and 3–23.4% among adult women, depending on the study design^1,2^. Untreated OSA is associated with an increased risk of motor vehicle accidents, cardiovascular disease^3^, atrial fibrillation^4^, and exacerbations of chronic airway diseases^5,6^.

Changes in the metabolome of OSA patients have thus far been studied in search for a biomarker or a set of biomarkers^7^. In most studies designed for finding a biomarker in OSA, the samples have been obtained in the morning after sleep, whereas studies, addressing the differences between the evening and morning samples or the samples taken during sleep, are scarce^8,9^.

Untargeted analysis of the metabolome in morning urine has been used on patients with OSA, which only partly reflects night-time metabolomic profile^7^. It is known that the results for untargeted metabolomic analyses are not always interchangeable between laboratories^10^, whereas targeted analysis, on the contrary, has shown better interlaboratory reproducibility^11^.

The metabolic pathways involved in OSA have not been thoroughly investigated yet. There have been no metabolomic studies that have investigated the metabolomic changes among patients with OSA during night-time or, more importantly, the dynamic changes taking place from the time of going to bed until waking up.

The substances examined were selected based upon the previous literature: the effects of sleep deprivation on amino acids and biogenic amine profile have been examined^12^, but there have been no studies on OSA and its effects on the serum profiles of amino acids and biogenic amines. Haptoglobin, ceruloplasmin and hsCRP have all been previously examined as possible biomarkers for obstructive sleep apnea^8,13,14^, however, prospective trials examining their biomarker potential have been lacking. General biochemical analyses were done to exclude a severe concomitant undiagnosed disease, which could alter the metabolome.

We hypothesized that assessing the metabolomic profile at multiple time points in the peripheral blood of OSA patients using a targeted approach could lead to better characterization of obstructive sleep apnea and its systemic effects in particular. This knowledge could improve the treatment and monitoring of OSA patients and lead to a more precise division into subgroups in the future. Together with precise clinical characterization and longitudinal follow-up this approach could lead to better risk assessment among OSA patients. The aim of our study was to assess the amino acid and biogenic amine profile in peripheral blood of OSA patients during multiple time points (evening, night-time, morning) in order to characterize the metabolomic differences between OSA patients and controls.

## Results

A total of 63 individuals were recruited, 31 with moderate-to-severe OSA and 32 controls (Table 1). The patients with OSA had significantly higher plasma concentrations of alanine aminotransferase (ALAT) (*p* = 0.006) and triglycerides (TG) (*p* = 0.02) (Table 1). The total sleep time and the time spent in different sleep phases in OSA patients did not differ statistically from that in the control individuals. There was a significant difference in leg movements per hour (*p* = 0.042) (Table 1). Additionally, 6 individuals had asthma (4 in the OSA and 2 in the control group) and 2 had allergic rhinitis (1 in the OSA group and 1 in the control group).

**Table 1:** Baseline characteristics of the study population, including serum biochemistry results (measured at 7:00 a.m.) and polysomnography data.

Variable	OSA patients (n = 31)	Controls (n = 32)	*p value
Male gender, n (%)	19 (61.3)	11 (34.4)	0.06
Age, years	57 (48–60)	48 (40.8–56.3)	0.03
BMI (kg/m^2^)	31.9 (27.7–35.5)	27.7 (23.9–29.4)	0.002
Neck circumference, cm	42.5 (39.8–44.3)	39.0 (36.0–41.0)	< 0.001
Active smokers, n (%)	10 (32.3)	8 (25)	0.71
STOP BANG score	5.0 (5.0–7.0)	3.5 (2.0–5.0)	< 0.001
ESS score	10.0 (5.0–12.0)	8.5 (5.75–11.25)	0.73
FBG (mmol/L)	6.0 (5.5–6.6)	5.5 (5.2–6.1)	0.003
ALAT (U/L)	23.0 (21.0–33.0)	20.0 (14.0–26.25)	0.006
ASAT (U/L)	22.0 (20.0–28.5)	22.0 (17.0–24.0)	0.12
Creatinine (μmol/L)	79.0 (70.5–87.5)	74.0 (62.8–79.0)	0.052
Urea (mmol/L)	5.0 (4.0–5.8)	4.9 (4.0–5.4)	0.62
Triglycerides (mmol/L)	1.6 (1.1–2.4)	1.2 (0.8–1.7)	0.02
AHI (/h)	31.1 (20.5–41.4)	6.2 (2.0–11.2)	< 0.001
TST (min)	450.5 (495.8–399.0)	432.2 (478.1–384.4)	0.67
N1 (min)	63.5 (90.3–33.7)	38.5 (72.6–30.0)	0.17
N2 (min)	211.1 (240.0–164.6)	226.8 (262.5–169.7)	0.36
N3 (min)	76.3 (104.9–32.2)	77.0 (102.4–59.6)	0.19
REM (min)	69.9 (96.3–54.7)	62.6 (79.8–51.9)	0.24
ODI < 90% (/h)	8.7 (2.3–18.3)	0.6 (0–2.8)	< 0.001
ODI ≥ 5% (/h)	3.4 (1.7–12.5)	0.3 (0.1–1.0)	< 0.001
Sleep-time leg movements (/h)	16.5 (6.9–35.8)	8.25 (5.5–14.4)	0.042
Arousal index (EEG arousals per hour of sleep)	16.9 (7.8–26.7)	10.9 (6.4–18.3)	0.055

Data are presented as median (IQR), unless otherwise specified.

*AHI* apnea–hypopnea index, *ALAT* alanine aminotransferase, *ASAT* aspartate aminotransferase, *BMI* body mass index, *EEG* electroencephalography, *ESS* Epworth sleepiness scale, *FBG* fasting blood glucose, *IQR* interquartile range, *N1–N3* time spent in N1, N2, and N3 sleep phases, respectively, *ODI* ≥ *5%* amount of oxygen desaturations of at least 5% per hour of sleep, *ODI* < *90%* amount of oxygen desaturations below 90% per hour of sleep, *OSA* obstructive sleep apnea, *REM* time spent in rapid eye movement (REM) sleep (sleep phases were scored according to the American Academy of Sleep Medicine guidelines^15^), *TST* total sleep time.

*Analyzed with the use of Mann–Whitney U test or Pearson’s Chi square test.

The TG values correlated significantly with that of body mass index (BMI) (ρ = 0.288, p = 0.022) and ALAT (ρ = 0.288, *p* = 0.006).

There were no significant differences in high-sensitivity C-reactive protein (hsCRP), haptoglobin and ceruloplasmin concentrations between OSA patients and controls (Supplementary Table S1).

**Figure 1:**
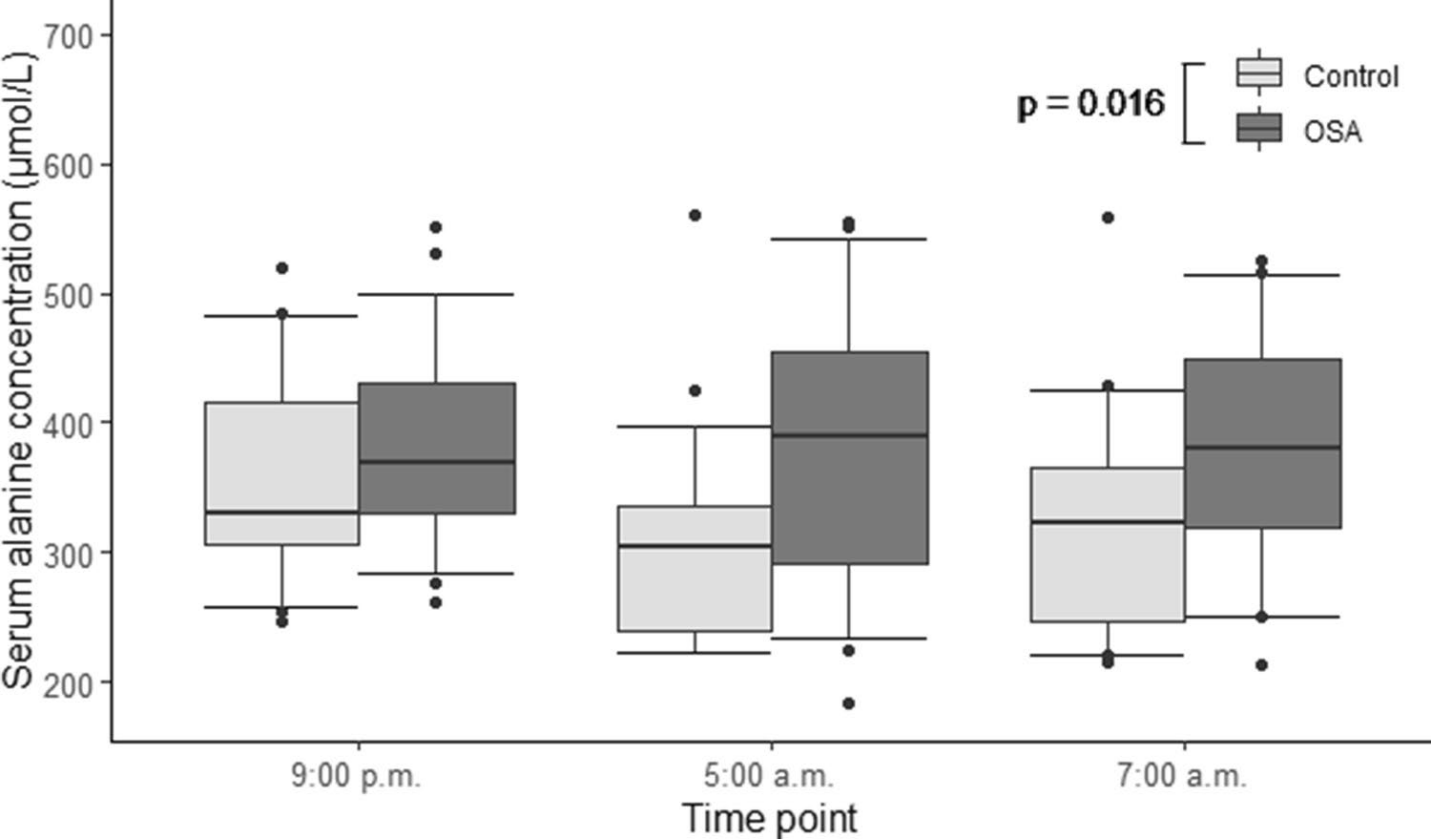
Serum alanine concentrations of patients with moderate-to-severe obstructive sleep apnea (OSA) (n = 31) and control individuals (n = 32). General linear model for repeated measures was used on rank transformed data. Alanine concentrations were significantly higher in OSA patients, compared to controls (*p* = 0.016).

**Figure 2:**
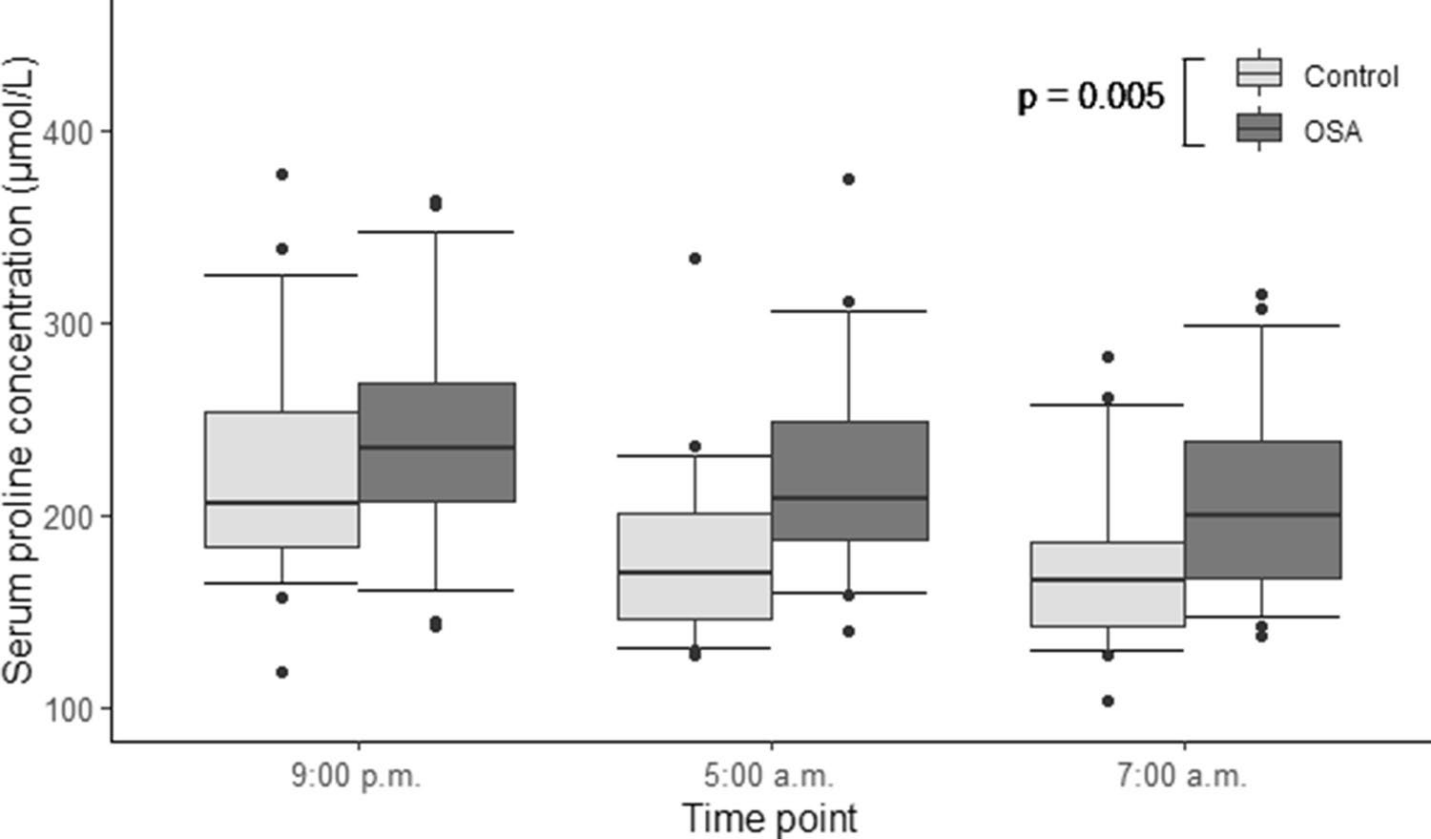
Serum proline concentrations of patients with moderate-to-severe obstructive sleep apnea (OSA) (n = 31) and control individuals (n = 32). General linear model for repeated measures was used on rank transformed data. Proline concentrations were significantly higher in OSA patients, compared to controls (*p* = 0.005).

**Figure 3:**
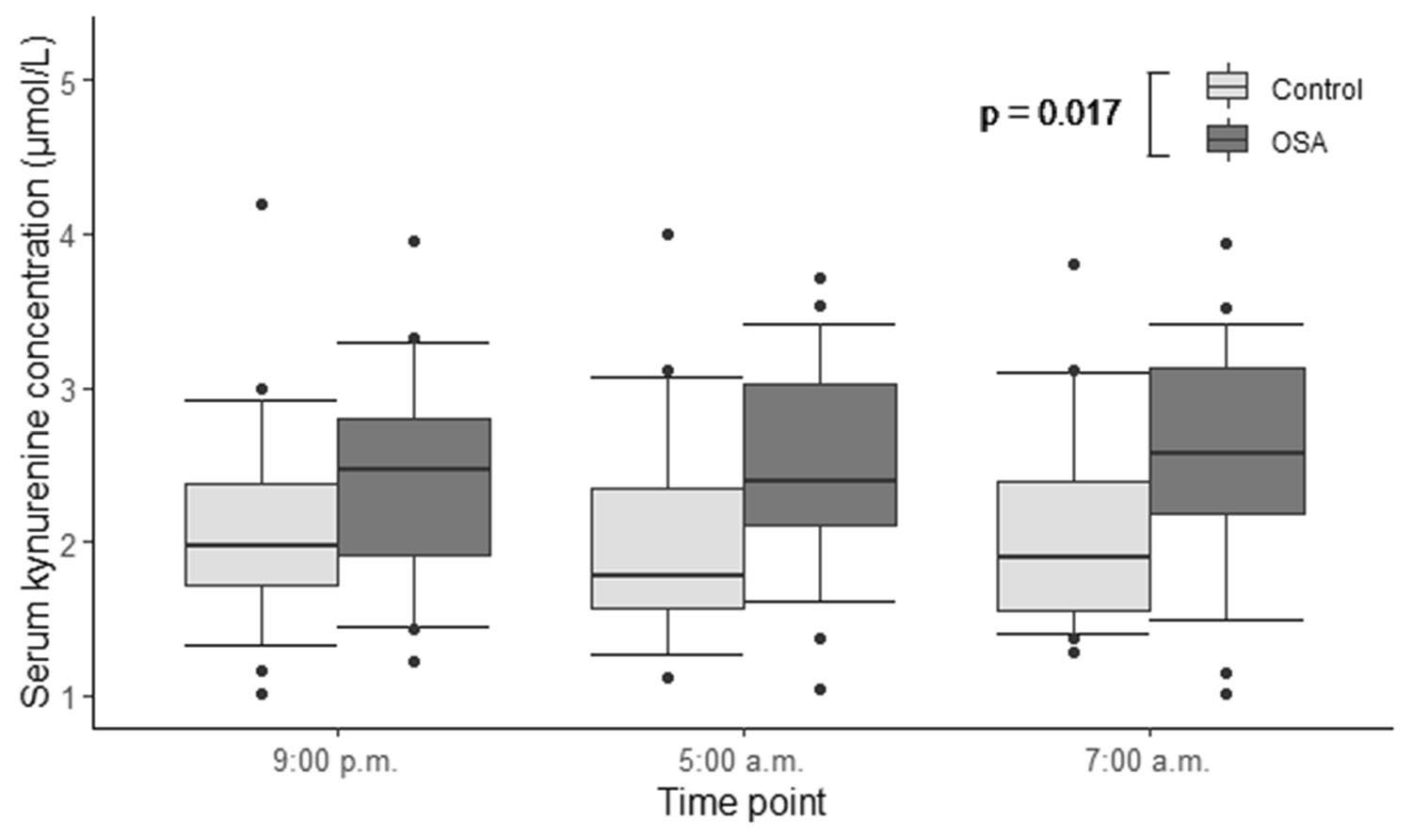
Serum kynurenine concentrations of patients with moderate-to-severe obstructive sleep apnea (OSA) (n = 31) and control individuals (n = 32). General linear model for repeated measures was used on rank transformed data. Kynurenine concentrations were significantly higher in OSA patients, compared to controls (*p* = 0.017).

A total of 29 amino acids and biogenic amines were analyzed. A comprehensive table containing the concentrations and *p*-values of every substance (hsCRP, haptoglobin, ceruloplasmin, amino acids and biogenic amines) that we analyzed at three different time points is presented in the online supplement (Supplementary Table S1). Significant elevations in the median serum concentrations of alanine (Ala) (*p* = 0.016, Fig. 1), proline (Pro) (*p* = 0.005, Fig. 2) and kynurenine (Kyn) (*p* = 0.017, Fig. 3) in OSA patients compared to controls were detected (Table 2).

**Table 2:** The results of overnight measurements of amino acids and biogenic amines in the sera of patients with moderate-to-severe obstructive sleep apnea (OSA) and control individuals at three time points: 9:00 p.m., 5:00 a.m. and 7:00 a.m. and the *p*-values of two separate ranked general linear model for repeated measures analyses.

Variable	Group	Time point			*p value	**p value
		9:00 p.m	5:00 a.m	7:00 a.m		
Alanine	Controls	331.0 (305.8–415.2)	304.0 (238.8–336.8)	322.0 (247.5–364.8)	0.016	0.30
	OSA	370.0 (330.0–430.0)	389.0 (290.5–455.0)	380.0 (318.5–449.5)		
Glutamine	Controls	738.0 (657.8–801.5)	707.0 (670.0–766.8)	699.0 (639.2–765.8)	0.70	0.025
	OSA	658.0 (613.5–716.0)	685.0 (630.0–800.5)	696.0 (648.5–773.5)		
Glycine	Controls	232.0 (204.8–263.2)	229.5 (204.0–272.0)	232.0 (207.0–273.0)	0.80	0.007
	OSA	200.0 (170.5–224.5)	221.0 (194.0–248.5)	233.0 (201.0–269.5)		
Phenylalanine	Controls	71.3 (62.9–78.7)	59.9 (54.1–65.5)	59.4 (52.6–64.5)	0.74	0.012
	OSA	69.2 (57.4–76.2)	69.5 (61.3–76.7)	66.2 (60.4–70.8)		
Proline	Controls	206.0 (184.2–253.2)	170.5 (146.8–201.5)	166.5 (142.5–186.8)	0.005	0.008
	OSA	235.0 (208.0–268.0)	209.0 (188.0–248.5)	200.0 (168.0–238.5)		
Serine	Controls	121.0 (107.8–130.0)	115.0 (100.7–127.2)	109.5 (94.6–136.0)	0.99	0.005
	OSA	102.0 (85.0–123.5)	111.0 (96.1–125.0)	111.0 (101.5–123.5)		
Threonine	Controls	127.0 (116.5–143.8)	109.5 (96.0–120.3)	110.5 (99.5–133.0)	0.67	0.001
	OSA	113.0 (97.5–124.0)	120.0 (92.8–135.0)	118.0 (93.0–132.5)		
Tryptophan	Controls	65.8 (53.9–74.2)	50.3 (44.8–62.2)	55.5 (47.6–59.2)	0.87	< 0.001
	OSA	59.6 (51.2–65.3)	62.1 (50.3–68.6)	59.7 (51.0–65.8)		
Kynurenine	Controls	1.97 (1.72–2.38)	1.78 (1.56–2.35)	1.90 (1.56–2.40)	0.017	0.001
	OSA	2.47 (1.92–2.81)	2.40 (2.15–2.98)	2.57 (2.19–3.13)		

Concentrations are expressed as μmol/L (interquartile range).

Statistical analysis has been performed with rank general linear model for repeated measures. The model was adjusted to body mass index, age, gender, current smoking status, oxygen desaturation index including only desaturations with at least 5% drop in oxygen saturation; oxygen desaturation index including only desaturations extending below 90% and serum contents of potassium, sodium, aspartate aminotransferase, alanine aminotransferase, urea, high-density lipoprotein cholesterol, low-density lipoprotein cholesterol and triglycerides.

*p values refer to between-group differences for serum concentrations of the variables between patients with moderate-to-severe OSA and control individuals.

**p values indicate time-dependent effects with time-by-group interactions for serum concentrations of the biomarkers referring only to the differences for the dynamics of the respective substances overnight between patients with moderate-to-severe OSA and control individuals.

**Figure 4:**
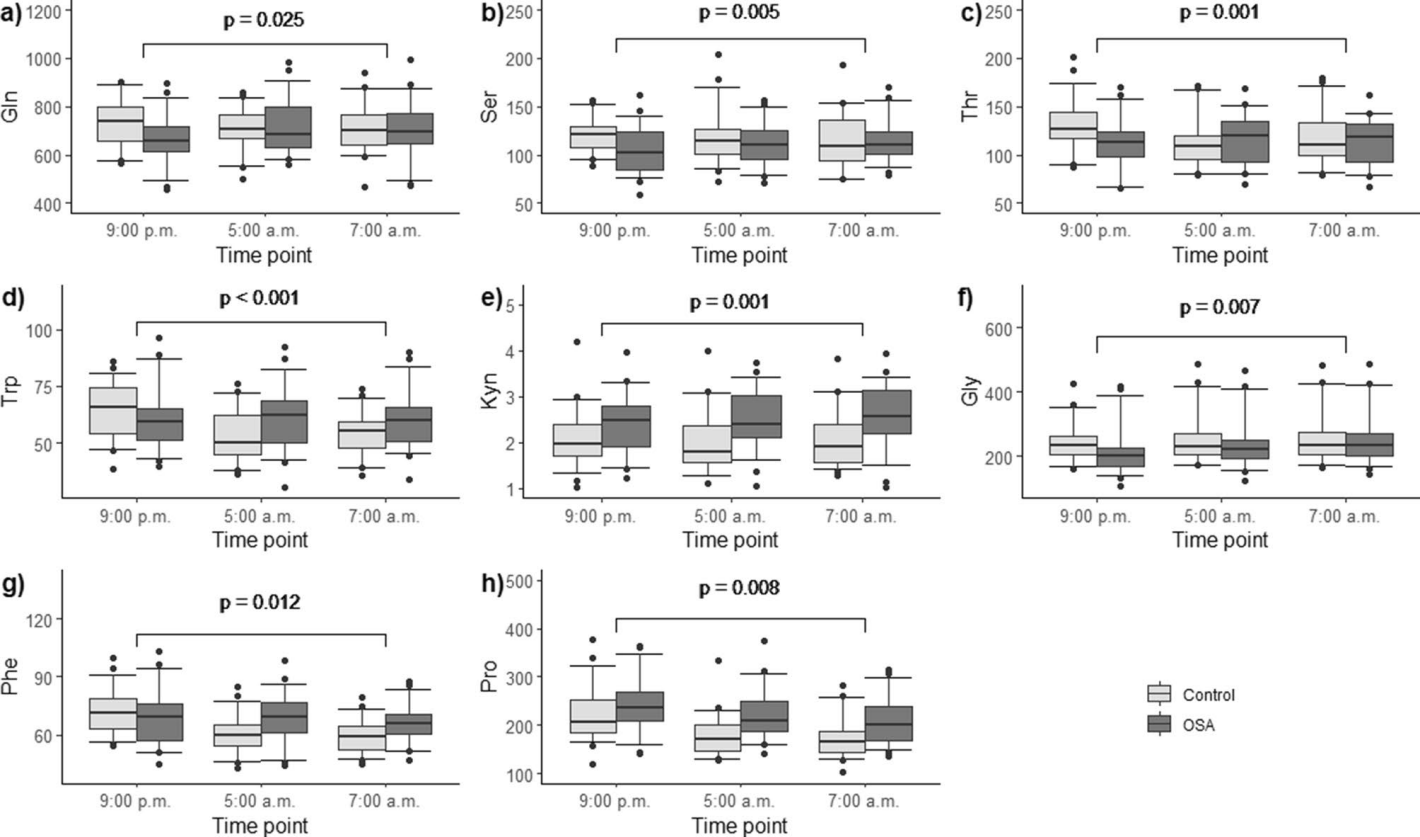
(a–h) Overnight dynamics of serum amino acid and biogenic amine concentrations in patients with moderate-to-severe obstructive sleep apnea (OSA) (n = 31), compared to controls (n = 32). Rank transformed data were subjected to general linear model for repeated measures. The differences for the overnight changes of the respective substances between patients with OSA and control individuals are shown. All data have been plotted as the concentration values (μmol/L) of a substance vs. the time point. (**a**) Glutamine, *p* = 0.025; (**b**) serine, *p* = 0.005; (**c**) threonine, *p* = 0.001; (**d**) tryptophan, *p* < 0.001; (**e**) kynurenine, *p* = 0.001; (**f**) glycine, *p* = 0.007; (**g**) phenylalanine, *p* = 0.012; (**h**) proline, *p* = 0.008.

**Figure 5:**
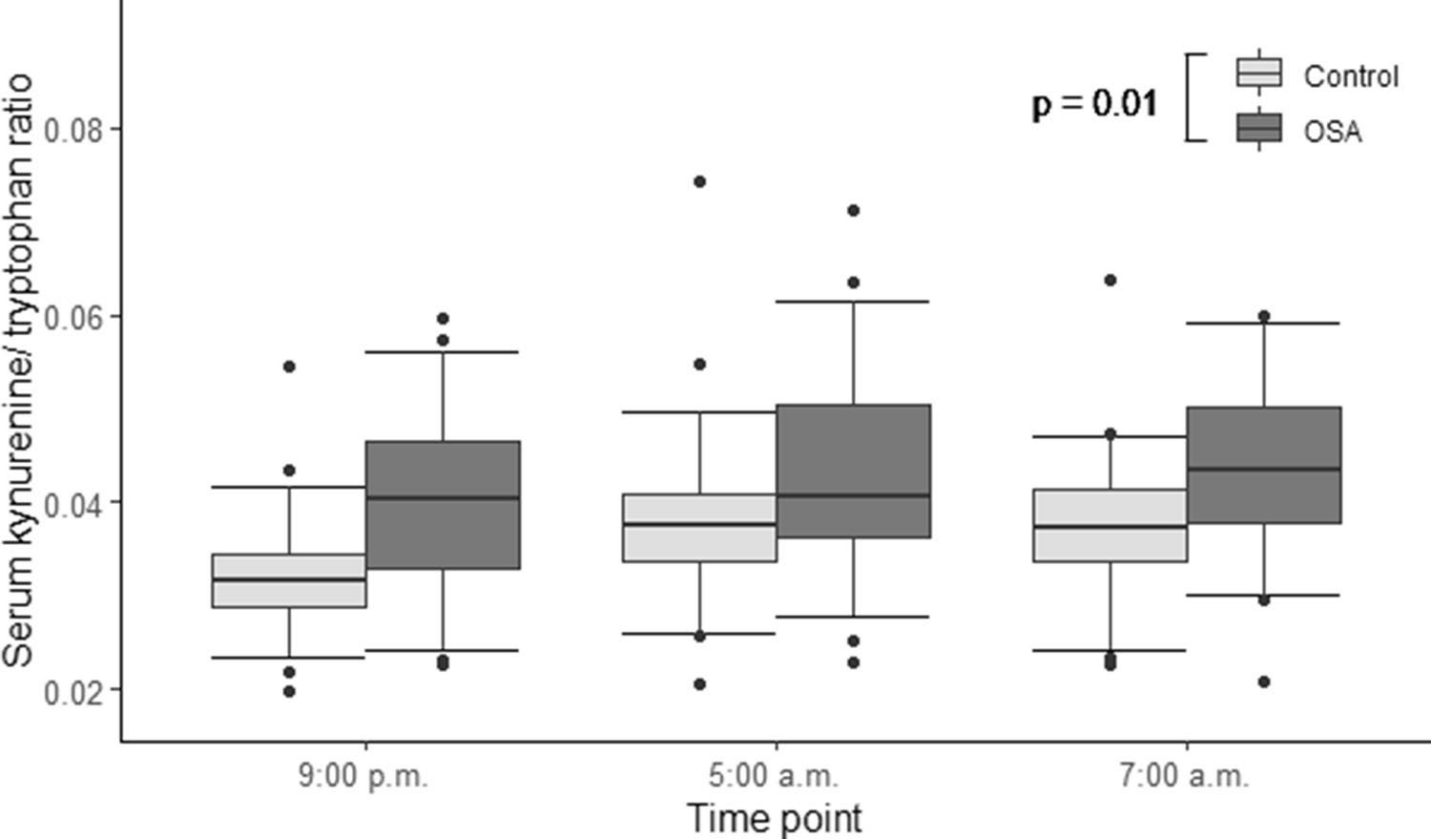
Kynurenine/tryptophan ratio in the serum of patients with moderate-to-severe obstructive sleep apnea (OSA) (n = 31) and control individuals (n = 32). Rank transformed data were subjected to general linear model for repeated measures. Kynurenine/tryptophan ratio in OSA patients was significantly higher, compared to controls (*p* = 0.01).

Furthermore, the within-subject tests revealed significant time-dependent effects along with significant time-by-group interactions for serum concentrations of certain substances referring to significantly different dynamics of these biomarkers overnight. In particular, the levels of glutamine (Gln) (*p* = 0.025), serine (Ser) (*p* = 0.005), threonine (Thr) (*p* = 0.001), tryptophan (Trp) (*p* < 0.001), Kyn (*p* = 0.001) and glycine (Gly) (*p* = 0.007) increased in the patients with OSA, whereas a fall occurred in the levels of the same biomarkers in controls (Fig. 4a–f, Table 2). Instead, the levels of phenylalanine (Phe) (*p* = 0.012) and Pro (*p* = 0.008) decreased slightly, compared to a steeper fall in controls (Fig. 4g,h, Table 2). Univariate modeling for Kyn/Trp showed the ratio being significantly heightened in OSA patients, compared to controls (*p* = 0.01) (Fig. 5). In addition, there were substances the concentration of which changed significantly overnight, but did not differ between OSA and control groups: isoleucine (*p* = 0.027) and asymmetric dimethylarginine (ADMA, *p* = 0.026) (Supplementary Table S1).

Finally, repeated measures’ analysis performed inside both groups indicated that the contents of several amino acids and biogenic amines decreased and/or increased significantly between different time points within the OSA group, as well as within the control group (Supplementary Table S2).

## Discussion

To our knowledge, this is the first study to address the metabolome of patients with OSA in peripheral blood serum measured at three different time points overnight in comparison to non-OSA control individuals. The differences in the Ala, Pro and Kyn median serum concentrations in moderate-to-severe OSA patient population were significantly different from that in control population.

In our current study, median Ala levels were increased in OSA patients, compared to controls. Ala is an essential component of the Cahill cycle^16^. Briefly, Ala is synthesized de novo in skeletal muscles by transamination of pyruvate. Consequently, in situations of augmented pyruvate availability, such as in skeletal muscles during exercise, increased release and/or accumulation of Ala in peripheral blood has been demonstrated^16^. Ala is taken up by the liver, where its carbon skeleton is reconverted to glucose. That glucose is again released into the blood circulation and taken up by muscles, where it is converted to pyruvate^16^. OSA is known to be associated with increased night-time muscle activity, due to either respiratory-related leg movements secondary to obstructive respiratory events^17^ or bathroom visits because of nocturia^3^. Although significantly higher in patients with OSA than in control individuals in our study, the night-time levels of Ala in OSA patients remained nearly stable and not deviating from the evening levels (Fig. 1). In contrast, the night-time Ala levels declined among the control individuals, compared to the levels in the evening. This finding could reflect the elevation in night-time muscle activity among OSA patients. To test this hypothesis, we did an additional correlation analysis to see, whether the nocturia, based on video recordings, and night-time leg movements are linked to the serum Ala concentration, but there was no significant correlation. As the significance of the maintained Ala levels throughout the night in patients with OSA remains obscure, further studies are needed to pinpoint the exact mechanisms as to why Ala concentration remains increased during the night-time in OSA.

Pro levels were increased in OSA patients, compared to controls (Fig. 2). The overnight change in Pro concentrations was also significantly different in OSA as compared to the control individuals: in OSA, the Pro levels stayed relatively steady during the night, whereas there was a significant drop in the control population (Fig. 4h). Moreover, the difference between the groups were most prominent during sleep (at 5 a.m.). It is well established that Pro constitutes about 10% of total amino acids in collagens and is thus essential for the biosynthesis of collagen^18^. The small intestine takes up considerable loads of dietary proline^18,19^. Tissue segments of pharyngeal muscles have been demonstrated to contain significantly more collagen I in OSA patients than in controls^20^. There are many hypotheses as to why collagen deposits in larger quantities in the pharyngeal muscles among OSA patients. A reaction to mechanical stress during snoring has been previously proposed^20^. Hence, this extra need for collagen synthesis during the night would serve as one explanation of the higher sleep-time Pro values.

Median Kyn levels were increased in OSA patients, compared to controls (Fig. 3). The overnight change in the rank-transformed values of Kyn was also significantly different in patients with OSA: the Kyn levels remained relatively unchanged during the night, but there was a significant drop in the Kyn levels in the control individuals (Fig. 4e). Kyn is formed from Trp by degradation, catalyzed by indoleamine 2,3-dioxygenase (IDO)^21^. This is the first step of the so-called kynurenine pathway, where Trp is catabolized to form nicotine amides and the vitamin niacin as end products^22,23^. IDO is stimulated by cytokines during inflammatory processes, such as interferon gamma, interferon alpha and tumor necrosis factor alpha (TNF-α)^21–25^. The circadian rhythm of interferon gamma has been previously demonstrated not to be significantly different among OSA patients and controls^26^. A recent meta-analysis demonstrated that TNF-α level in patients with OSA was significantly (1.77 times) higher than in the control group^27^. Kyn/Trp represents a measure of IDO activity^24,28^. The supplementary analysis of Kyn/Trp confirmed that the Kyn/Trp values were significantly higher in the patients with OSA than in the controls and remained higher throughout the whole night as well (Fig. 5). This finding implies that IDO activity could be increased in OSA during night. The increased IDO activity in turn could be due to increased systemic inflammation (via TNF-α) in OSA patients.

The overnight elevations of Ser and Thr have been previously documented in urine specimens of simple snorers, compared to control population^7^. However Ser values in urine have been previously shown to decrease in OSA patients compared to controls^7^. The significant change in the overnight dynamics of Trp and Kyn shown in our current study has not been demonstrated previously. The morning fasting serum levels of Kyn and Trp have been investigated in obstructive sleep apnea, but no significant differences between OSA and controls were found^29^. This further underlines the importance of assessing the levels of potential pathophysiological biomarkers in OSA at different time points. The significant overnight changes in the concentrations of Gly, Phe and Pro in OSA patients compared to controls have not been previously described.

In our current study, the levels of ALAT and TG were significantly elevated in OSA patient group, compared to the control population in the morning fasting blood samples. The rise of ALAT and TG concentrations could be explained by the increased BMI of the OSA patients. It has been shown previously that increased TG levels are positively correlated with increased BMI^30^. Increased BMI is also associated with non-alcoholic fatty liver disease (NAFD)^31,32^. Increased ALAT levels are shown to occur with NAFD as well^32^. As the TG concentrations correlated positively with that of ALAT, as well as with BMI in OSA, a possibility exists that the increased levels of ALAT could have a link with increased BMI via NAFD in patients with OSA.

The strengths of this study were the measurement of the metabolome at different time points, usage of strict exclusion criteria and obtaining the analyses via venipuncture. PSG recording on the same night as blood sampling is important for precise linking of molecular phenotype to sleep quality. Venipuncture was thought of as clinically more feasible, as the placing of an indwelling catheter would have made it more difficult to implement our findings into clinical practice. It was also considered that the effects of heparin, used for flushing, and catheter induced local inflammation^33^ might have been important metabolome changing factors, thus obscuring the data. Measuring of the metabolome at different time points was done to minimize the effects of random or clock-time dependent factors on the metabolome. Blood sampling was scheduled to take place during sleep, in order to characterize sleep-time changes.

Among the limitations of our study, the sample size of 63 participants is relatively small. However, this limitation was attenuated by samples taken at multiple time point. In our study the patients with OSA were significantly older, had greater BMI and neck circumference, higher STOP-BANG scores and fasting blood glucose (FBG) values. These differences, however, do not represent a bias but were rather due to the fact that OSA itself is more frequent with higher BMI and older age^3^ and because the study participants were recruited consequentially.

In conclusion, we have found that the concentrations of certain amino acids and biogenic amines including Ala, Pro and Kyn are elevated in the peripheral blood of OSA patients between 9 p.m. and 7 a.m. The profile of several amino acids and biogenic amines changed significantly when measured in OSA patients at multiple time points during the night and compared to control population. The differences in the concentrations of Ala, Pro, and Kyn are especially pronounced during the night-time and early morning blood samples.

## Methods

We conducted a single-center prospective observational study to compare the metabolomic blood profiles of moderate-to-severe OSA patients and controls. This study was performed in accordance with the Declaration of Helsinki and the study protocol was approved by the Tallinn Medical Research Ethics Committee (decision number 2270). Written informed consent was obtained from each participant.

### Study population

Adult individuals were recruited from the Department of Psychiatry of the Tartu University Hospital, where they were referred for a standard polysomnography (PSG). Exclusion criteria included acute illness, defined as the presence of symptoms of acute infection, treatment with continuous positive airway pressure (CPAP) whenever during the last 6 months, current diagnosis of a chronic illness like heart failure in New York Heart Association (NYHA) class III–IV, chronic kidney disease stage IV–V, degenerative cerebrovascular disease, other neurological disease, liver disease, pulmonary disease with oxygen saturation below 93%, active malignancy, autoimmune disease, type I and type II diabetes, and treatment with drugs known to affect metabolome including systemic corticosteroids and hormonal contraceptives. The recruitment period lasted from April 2018 to September 2019.

### Polysomnography

We used standard PSG recording^15^, which included video monitoring, electroencephalography, eletctrooculography, chin and leg electromyography, airflow, thoracoabdominal bands, snoring sensor, body position, electrocardiography, and oxygen saturation. PSG was performed with use of either an Embletta MPR (Natus Medical Inc., San Carlos, CA, USA) or a NOX A1 (Nox Medical, Reykjavik, Iceland) as these were routine standards in our center. PSG data were manually scored by European Sleep Research Society certified sleep technicians according to American Academy of Sleep Medicine (AASM) PSG scoring guidelines^15^.

The clinical diagnosis of OSA was based on obstructive respiratory events alone as per international recommendations^34^. The central apneas were also included in the AHI calculation and AHI was further used to determine the severity class of OSA (mild, moderate, severe). In every patient, there were altogether more obstructive than central respiratory events and no patient in our study had central sleep apnea or Cheyne–Stokes breathing diagnosed.

The subjects were divided into moderate-to-severe OSA group and control group based on the PSG findings and more specifically, on the AHI^3^. Participants with AHI ≥ 15/h were termed as moderate-to-severe OSA group and those with AHI < 15/h were included in control group.

### Blood sample collection

Blood samples for metabolomics and clinical biochemistry were collected simultaneously with PSG recording via standard peripheral venipuncture at 3 different time points: 9:00 p.m.; 5:00 a.m.; 7:00 a.m. These time points were carefully chosen to characterize in the best way the sleep-time changes among the study population on one hand and to avoid interfering with the normal sleep structure on the other. We used BD Vacutainer^®^ silicone coated (REF 367614, Beckton Dickinson, Franklin Lakes, NJ, USA) extraction tubes for collection of serum samples for metabolomics. Clotting was allowed for 30 min at room temperature and the samples were then centrifuged at 1500*g* for 15 min at 4 °C. The resulting sera were aliquoted and frozen at − 80 °C until further analysis. The entire process was completed within 60 min of blood extraction.

Analyses for markers of systemic inflammation (hsCRP) and possible proteomic OSA biomarkers (haptoglobin, ceruloplasmin)^14^ were taken at all 3 time points mentioned above. Other clinical parameters like sodium (Na), potassium (K), creatinine, urea, ALAT, aspartate aminotransferase (ASAT), cholesterol, LDL (low-density lipoprotein) cholesterol, HDL (high-density lipoprotein) cholesterol, TG were analyzed once at 7:00 a.m. FBG was also measured at 7:00 p.m. For the clinical biochemistry sample collection, we used BD Vacutainer^®^ Heparin (REF 368886, Beckton Dickinson, Franklin Lakes, NJ, USA) extraction tubes.

### Analysis of metabolites

Amino acids and biogenic amines were measured in sera using liquid chromatography-mass-spectrometry [(LC)–MS/MS] techniques. For measuring of the metabolite levels, we used a targeted approach: previously validated AbsoluteIDQ™ p180 kit (BIOCRATES Life Sciences AG, Innsbruck, Austria)^11^ was used to determine the serum levels of different amino acids and biogenic amines. Frozen sera were thawed at room temperature and subsequently analyzed on mass-spectrometry QTRAP 4500 (Sciex, Framingham, MA, USA), linked to a high-performance liquid chromatography (HPLC) (Agilent 1260 series, Agilent Technologies, Waldbronn, Germany). Preparation of samples and all measurements were done as described by the manufacturer in the test kit manual UM-P180; the specific details are described previously^35^. Concentrations of the metabolites were calculated automatically by the MetIDQ™ software (BIOCRATES Life Sciences AG). The AbsoluteIDQ™ test kit has internal standards and includes quality control samples, which were evaluated, deviations were noted and corrections were done when necessary in the MetIDQ software before start of data analysis. Analytes that were considered invalid by the software were excluded from later statistical analysis. The kit itself has been estimated to have inter-laboratory coefficients of variation below 10% for most metabolites relevant for the current study^11^.

### Analysis of samples for biochemistry

In case of this analysis set, plasma samples were obtained and analyzed at the United Laboratories of the Tartu University Hospital. ALAT, ASAT and Urea levels were measured fully automatically using kinetic photometric method. HDL-cholesterol, LDL-cholesterol, total cholesterol and TG were measured fully automatically using enzymatic colorimetric method. Sodium and potassium concentrations were measured fully automatically using ion-selective electrodes. Creatinine was measured fully automatically using an enzymatic method. hsCRP was measured fully automatically using particle-enhanced immunoturbidimetric assay. All the substances mentioned above were measured using Cobas^®^ 6000 c501 analyzer (Roche Diagnostics, F. Hoffmann-La Roche AG, Basle, Switzerland), which has been previously validated^36^. Ceruloplasmin and haptoglobin were measured fully automatically using immunoturbidimetric assay. These two substances were measured using Cobas^®^ Integra 400 analyzer (Roche Diagnostics), which has been previously validated^37^.

### Statistical analysis

We used ranked general linear model for repeated measures to detect significant differences in the serum contents of amino acids and biogenic amines between patients with OSA and control individuals, as well as to unravel significant changes occurring overnight. To achieve the best fitting model, we included all independent demographic, clinical and laboratory covariates first and used backward elimination that resulted in BMI, age, gender, current smoking status, oxygen desaturation index that included only desaturations with at least 5% drop in oxygen desaturation (ODI ≥ 5%), oxygen desaturation index that included only desaturations extending below 90% (ODI < 90%) and serum contents of K, Na, ASAT, ALAT, urea, HDL-cholesterol, LDL cholesterol and TG remaining in the final model as covariates the outcomes were adjusted for. Afterwards, a supplementary univariate comparison between the patients and control individuals for the kynurenine-tryptophan (Kyn/Trp) values was performed, also adjusted for the same covariates. All variables were rank transformed prior to all analyses, as were the outcome variables (i.e. the serum contents of amino acids and biogenic amines) assigned standardized ranks over the three time points. Fischer’s least significant difference method was used for correction for multiple comparisons.

The pre-analysis characteristics were compared between the patients with moderate-to-severe OSA and controls using Mann–Whitney U test. Spearman correlation analysis was used for assessing correlation between the obtained findings. All data are presented as medians and interquartile ranges (IQR) or numbers (%). The statistical analyses were performed using SPSS software, version 20.0 (IBM Co, NY, USA). On all figures, the boxplots were constructed as follows: central line = median; upper box limit = 3rd quartile; lower box limit = 1st quartile; upper whisker limit = 95th percentile; lower whisker limit = 5th percentile. Black dots represent outliers.

## Supplementary Information

Supplementary Information 1.
