# Supplementary material for: The effect of obstructive sleep apnea on peripheral blood amino acid and biogenic amine metabolome at multiple time points overnight
Source: Sci Rep. 2021 May 24;11:10811. doi: 10.1038/s41598-021-88409-y (PMC8144378; doi:10.1038/s41598-021-88409-y)
Supplement: Supplementary file 1 — Supplementary Information 1. [file 41598_2021_88409_MOESM1_ESM.pdf]

Supplementary Appendix

This appendix has been provided by the authors to give readers additional information about their work.

Supplement to: „The effect of obstructive sleep apnea on peripheral blood amino acid and biogenic amine metabolome at multiple time points overnight“

Authors: Ott Kiens [1,2], Egon Taalberg [3,4], Viktoria Ivanova [2], Ketlin Veeväli [5], Triin Laurits [5], Ragne Tamm [5], Aigar Ottas[3,4], Kalle Kilk [3,4], Ursel Soomets [3,4], Alan Altraja [1,2]

Affiliations:

1- Department of Pulmonary Medicine, University of Tartu, Estonia
2- Lung Clinic, Tartu University Hospital, Estonia
3- Institute of Biomedicine and Translational Medicine, University of Tartu, Estonia
4- Centre of Excellence for Genomics and Translational Medicine, University of Tartu, Estonia
5- Psychiatry Clinic, Tartu University Hospital, Estonia

Corresponding Author: Ott Kiens, Tartu University Lung Clinic, Riia 167, 50411, Tartu, Estonia, ott.kiens@kliinikum.ee

Supplementary table S1. The results of overnight measurements of hsCRP, haptoglobin, ceruloplasmin, amino acids and biogenic amines in the sera of patients with moderate-to-severe obstructive sleep apnea (OSA) (n = 31) and control individuals (n = 32) at three time points: 9:00 p.m., 5:00 a.m. and 7:00 a.m. Positive values represent increase and negative ones show decrease in the concentrations between the time points. Concentrations are expressed as µmol/L unless otherwise stated. Interquartile ranges are provided in parentheses.

Variable	Time point			*p-value	**p-value	***p-value
	9:00 p.m.	5:00 a.m.	7:00 a.m.			
hsCRP (mg/L)				0.25	0.56	0.85
Controls	1.2 (1.9–0.6)	1.1 (1.7–0.4)	1.0 (1.7–0.5)			
OSA	2.0 (4.1–1.1)	1.7 (4.1–0.8)	1.6 (4.1–0.9)			
Haptoglobin (g/L)				0.57	0.27	0.48
Controls	0.97 (1.10–0.77)	0.92 (1.04–0.71)	0.93 (1.06–0.74)			
OSA	1.27 (0.91–1.68)	1.23 (1.60–0.85)	1.21 (1.58–0.81)			
Ceruloplasmin (g/L)				0.99	0.23	0.49
Controls	0.25 (0.29–0.23)	0.23 (0.25–0.21)	0.23 (0.26–0.21)			
OSA	0.24 (0.27–0.23)	0.22 (0.25–0.20)	0.23 (0.25–0.21)			
Alanine				0.016	0.30	0.77
Controls	331.0 (415.2–305.8)	304.0 (336.8–238.8)	322.0 (364.8–247.5)			

OSA	370.0 (430.0–330.0)	389.0 (455.0–290.5)	380.0 (449.5–318.5)			
Arginine				0.57	0.13	0.88
Controls	114.0 (117.5–102.0)	102.5 (113.8–97.6)	108.0 (119.8–95.7)			
OSA	121.0 (136.5–99.4)	113.0 (140.0–104.5)	122.0 (135.0–97.8)			
Asparagine				0.80	0.72	0.66
Controls	47.3 (51.5–39.6)	44.8 (49.2–37.3)	43.3 (48.0–35.2)			
OSA	42.5 (45.9–36.1)	42.8 (46.0–37.9)	40.5 (48.4–36.8)			
Aspartate				0.60	0.77	0.50
Controls	11.9 (15.9–9.5)	10.6 (12.1–8.1)	9.3 (10.4–8.3)			
OSA	13.1 (15.3–9.5)	11.3 (13.7–8.3)	9.6 (12.4–8.1)			
Citrulline				0.23	0.91	0.36
Controls	27.7 (31.5–22.8)	28.6 (30.5–24.8)	25.2 (29.6–21.9)			
OSA	28.7 (37.1–21.3)	28.9 (34.8–22.9)	28.7 (33.8–23.3)			
Glutamine				0.70	0.025	0.99
Controls	738.0 (801.5–657.8)	707.0 (766.8–670.0)	699.0 (765.8–639.2)			
OSA	658.0 (716.0–613.5)	685.0 (800.5–630.0)	696.0 (773.5–648.5)			

Glutamate				0.15	0.55	0.10
Controls	45.0 (58.9–26.9)	40.3 (65.8–28.7)	40.0 (49.6–31.2)			
OSA	64.0 (84.7–45.8)	62.0 (81.9–50.2)	65.5 (85.3–48.1)			
Glycine				0.80	0.007	0.300
Controls	232.0 (263.2–204.8)	229.5 (272.0–204.0)	232.0 (273.0–207.0)			
OSA	200.0 (224.5–170.5)	221.0 (248.5–194.0)	233.0 (269.5–201.0)			
Histidine				0.17	0.46	0.48
Controls	84.3 (93.3–79.1)	80.5 (93.0–75.5)	82.0 (89.5–74.2)			
OSA	84.6 (91.6–77.1)	89.0 (96.4–83.0)	87.0 (97.5–81.9)			
Isoleucine				0.78	0.58	0.027
Controls	90.8 (123.2–85.3)	76.3 (91.4–67.5)	67.8 (85.4–62.4)			
OSA	96.0 (111.0–77.5)	83.5 (93.5–75.4)	83.1 (94.2–68.1)			
Leucine				0.40	0.33	0.24
Controls	160.5 (204.8–135.0)	140.5 (169.5–123.8)	135.0 (156.5–113.8)			
OSA	163.0 (192.0–129.0)	156.0 (183.5–139.5)	161.0 (177.0–134.5)			
Lysine				0.79	0.30	0.36

Controls	186.0 (231.2–170.8)	185.5 (207.5–157.2)	187.0 (223.2–169.0)			
OSA	201.0 (249.5–173.5)	211.0 (242.5–175.5)	221.0 (245.0–181.0)			
Methionine				0.57	0.25	0.13
Controls	25.1 (28.5–20.8)	21.0 (23.6–18.4)	22.1 (25.1–18.5)			
OSA	20.0 (25.9–18.6)	22.9 (26.9–18.9)	23.3 (25.8–20.6)			
Ornitine				0.65	0.09	0.42
Controls	69.7 (86.5–58.3)	62.3 (70.5–57.6)	57.8 (64.4–51.3)			
OSA	80.9 (96.7–60.9)	72.0 (78.7–56.8)	71.6 (79.1–55.9)			
Phenylalanine				0.74	0.012	0.27
Controls	71.3 (78.7–62.9)	59.9 (65.5–54.1)	59.4 (64.5–52.6)			
OSA	69.2 (76.2–57.4)	69.5 (76.7–61.3)	66.2 (70.8–60.4)			
Proline				0.005	0.008	0.66
Controls	206.0 (253.2–184.2)	170.5 (201.5–146.8)	166.5 (186.8–142.5)			
OSA	235.0 (268.0–208.0)	209.0 (248.5–188.0)	200.0 (238.5–168.0)			
Serine				0.99	0.005	0.58
Controls	121.0 (130.0–107.8)	115.0 (127.2–100.7)	109.5 (136.0–94.6)			

OSA	102.0 (123.5–85.0)	111.0 (125.0–96.1)	111.0 (123.5–101.5)			
Threonine				0.67	0.001	0.80
Controls	127.0 (143.8–116.5)	109.5 (120.3–96.0)	110.5 (133.0–99.5)			
OSA	113.0 (124.0–97.5)	120.0 (135.0–92.8)	118.0 (132.5–93.0)			
Tryptophan				0.87	< 0.001	0.64
Controls	65.8 (74.2–53.9)	50.3 (62.2–44.8)	55.5 (59.2–47.6)			
OSA	59.6 (65.3–51.2)	62.1 (68.6–50.3)	59.7 (65.8–51.0)			
Tyrosine				0.20	0.08	0.67
Controls	63.3 (75.6–53.6)	49.6 (61.6–46.6)	51.9 (61.1–46.1)			
OSA	62.5 (80.2–47.4)	62.6 (72.8–53.5)	62.4 (73.5–55.2)			
Valine				0.43	0.35	0.48
Controls	247.5 (282.5–205.5)	221.0 (267.2–184.2)	218.5 (250.2–199.8)			
OSA	265.0 (302.5–215.5)	249.0 (274.5–223.0)	240.0 (277.0–214.0)			
ADMA				0.34	0.10	0.026
Controls	0.435 (0.545–0.357)	0.479 (0.548–0.403)	0.474 (0.542–0.391)			
OSA	0.437 (0.511–0.346)	0.487 (0.568–0.430)	0.491 (0.535–0.422)			

Creatinine				0.83	0.34	0.45
Controls	77.0 (86.6–64.6)	74.7 (79.8–62.3)	73.3 (78.8–62.1)			
OSA	80.4 (89.0–70.0)	79.7 (86.5–69.0)	78.7 (86.1–70.4)			
Kynurenine				0.017	0.001	0.48
Controls	1.97 (2.38–1.72)	1.78 (2.35–1.56)	1.90 (2.40–1.56)			
OSA	2.47 (2.81–1.92)	2.40 (2.98–2.15)	2.57 (3.13–2.19)			
Serotonin				0.29	0.63	0.89
Controls	0.385 (0.773–0.138)	0.429 (0.715–0.124)	0.326 (0.693–0.097)			
OSA	0.521 (0.788–0.347)	0.498 (0.687–0.363)	0.509 (0.635–0.349)			
Taurine				0.66	0.67	0.32
Controls	86.4 (98.8–73.1)	71.4 (90.1–59.9)	68.1 (83.3–58.8)			
OSA	88.9 (99.1–75.4)	78.3 (93.0–68.4)	74.4 (88.7–64.3)			
Kyn/Trp ratio				0.01	0.87	0.024
Controls	0.032 (0.034–0.029)	0.037 (0.041–0.034)	0.037 (0.041–0.034)			
OSA	0.040 (0.047–0.033)	0.041 (0.050–0.036)	0.043 (0.050–0.038)			

Statistical analysis has been performed with rank general linear model for repeated measures. The model was adjusted to body mass index, age, gender, current smoking status, oxygen desaturation index including only desaturations with at least 5% drop in oxygen saturation; oxygen desaturation index including only desaturations extending below 90% and plasma contents of potassium, sodium, aspartate aminotransferase, alanine aminotransferase, urea, high-density lipoprotein cholesterol, low-density lipoprotein cholesterol and triglycerides.

*p-values refer to between-group differences for serum concentrations of the variables between patients with moderate-to-severe OSA and control individuals.

**p-values indicate time-dependent effects with time-by-group interactions for serum concentrations of the biomarkers referring only to the differences for the dynamics of the respective substances overnight between patients with moderate-to-severe OSA and control individuals.

***p-values indicate shifts in concentrations of the variables overnight. This analysis does not take into account the differences between OSA and control groups.

ADMA asymmetric dimethylarginine, *hsCRP* high-semsitivity C-reactive protein.

Supplementary table S2. The changes in serum concentrations of hsCRP, haptoglobin, ceruloplasmin, amino acids and biogenic amines between different time points (9:00 p.m., 5:00 a.m. and 7:00 a.m.) among patients with moderate-to-severe obstructive sleep apnea (OSA) (n = 31) and among control individuals (n = 32). Data are presented as μmol/L unless otherwise stated. Interquartile ranges are provided in parentheses.

	Change					
	From 9:00 p.m. to 5:00 a.m.		From 9:00 p.m. to 7:00 a.m.		From 5:00 a.m. to 7:00 a.m.	
Variable		p-value		p-value		p-value
hsCRP (mg/L)						
Controls	0.2 (0.1–0.3)	0.005	0.2 (0.0–0.4)	0.013	1.0 (0.5–1.7)	0.56
OSA	0.3 (-0.1–0.5)	0.43	0.2 (-0.1–0.6)	0.11	0 (-0.1–0.2)	0.16
Haptoglobin (g/L)						
Controls	0.05 (0.0–0.1)	0.001	0.04 (-0.01–0.07)	0.006	-0.01 (-0.03–0.0)	0.07
OSA	0.05 (0.01–0.03)	< 0.001	0.08 (0.01–0.12)	0.005	-0.01 (-0.03–0.02)	0.25
Ceruloplasmin (g/L)						
Controls	0.02 (0.01–0.03)	< 0.001	0.02 (0.0–0.03)	< 0.001	0.0 (0.0–0.0)	< 0.001
OSA	0.02 (0.01–0.03)	< 0.001	0.02 (0.01–0.03)	< 0.001	0.0 (0.0–0.0)	0.27

Alanine						
Controls	66.0 (4.5–111.0)	0.005	34.0 (-20.0–89.5)	0.10	-7.5 (-46.8–2.5)	0.009
OSA	9.0 (-76.0–61.0)	0.72	12.0 (-64.0–54.5)	0.78	-11.0 (-33.5–31.5)	0.83
Arginine						
Controls	15.0 (24.8–-5.3)	0.029	11.5 (24.8–-8.3)	0.11	-1.0 (10.6–-15.6)	0.26
OSA	-2.1 (15.5–-12.5)	0.77	4.6 (12.0–5.3)	0.82	2.0 (17.9–-6.5)	0.62
Asparagine						
Controls	3.3 (-3.4–10.0)	0.15	5.1 (-0.1–8.2)	0.011	0.5 (-2.4–4.7)	0.26
OSA	-2.8 (-7.2–4.2)	0.42	-2.5 (-6.4–5.8)	0.96	1.6 (-1.4–4.8)	0.36
Aspartate						
Controls	1.7 (-0.6–4.5)	0.01	2.5 (0.8–6.6)	<0.001	0.9 (-1.3–2.1)	0.09
OSA	1.4 (-0.4–5.7)	0.15	2.6 (-0.5–4.1)	0.003	0.4 (-0.8–2.3)	0.06
Citrulline						
Controls	-0.4 (-2.9–5.2)	0.89	2.2 (-3.0–5.3)	0.31	0.7 (-1.0–4.4)	0.15
OSA	-0.3 (-3.4–3.4)	0.36	-0.1 (-4.5–3.4)	0.85	0.2 (-2.8–3.8)	0.35
Glutamine						

Controls	45.0 (-34.3–97.3)	0.49	20.5 (-13.8–97.0)	0.19	23.0 (-80.0–74.5)	0.56
OSA	-44.0 (-144.5–32.0)	0.051	-52.0 (-117.5–31.0)	0.009	1.0 (-59.5–89.5)	0.89
Glutamate						
Controls	-2.5 (-10.3–6.2)	0.75	2.3 (-14.3–12.2)	0.34	-0.3 (-7.4–11.6)	0.51
OSA	-3.8 (-10.4–14.9)	0.58	-0.6 (-13.8–13.6)	0.95	-2.4 (-11.75–5.75)	0.62
Glycine						
Controls	-6.5 (-20.0–16.0)	0.85	-16.5 (-26.8–6.5)	0.45	1.0 (-21.8–14.0)	0.60
OSA	-22.0 (-42.0–1.0)	0.008	-30.0 (-48.5–-15.5)	<0.001	-6 (-26–11)	0.16
Histidine						
Controls	3.9 (-1.3–10.9)	0.07	5.6 (-1.5–8.3)	0.18	-0.4 (-8.7–5.0)	0.88
OSA	-5.0 (-14.4–7)	0.13	-5.4 (-10.5–5.3)	0.099	-0.8 (-5.4–6.3)	0.85
Isoleucine						
Controls	17.4 (4.5–40.2)	< 0.001	26.3 (13.4–38.3)	< 0.001	6.4 (-3.3–13.2)	0.023
OSA	11.5 (-10.0–24.3)	0.028	9.4 (-6.6–28.0)	0.013	5.4 (-2.3–10.2)	0.24
Leucine						
Controls	17.5 (0.8–54.0)	0.001	31.5 (7.8–55.0)	0.001	11.5 (-9.3–21.5)	0.10

OSA	-1.0 (-33.0–42.5)	0.99	0.0 (-29.0–52.0)	0.75	8.0 (-8.0–21.0)	0.59
Lysine						
Controls	9.0 (-6.3–26.5)	0.03	9.0 (-20.0–28.0)	0.78	-11.0 (-33.0–6.3)	0.09
OSA	-10.0 (-30.0–38.0)	0.58	2.0 (-35.0–28.5)	0.26	-6.0 (-20.5–19.5)	0.55
Methionine						
Controls	3.3 (0.9–6.6)	< 0.001	2.1 (-0.9–6.9)	0.035	-0.6 (-2.9–1.8)	0.18
OSA	-0.6 (-6.4–6.0)	0.50	-3.4 (-5.6–2.8)	0.27	-0.9 (-2.5–3.1)	0.55
Ornitine						
Controls	7.2 (3.3–19.9)	0.005	12.4 (3.2–26.2)	< 0.001	4.1 (-5.3–13.8)	0.03
OSA	13.0 (-4.4–20.2)	0.035	11.0 (-0.8–19.2)	0.01	2.0 (-5.1–5.3)	0.62
Phenylalanine						
Controls	11.6 (6.0–16.1)	< 0.001	12.3 (4.5–19.3)	< 0.001	-0.4 (-4.4–5.2)	0.58
OSA	1.5 (-9.8–9.7)	0.85	0.1 (-7.9–13.5)	0.50	2.1 (-2.4–6.9)	0.23
Proline						
Controls	46.5 (23.0–61.3)	< 0.001	41.0 (27.0–70.3)	< 0.001	10.5 (-17.5–20.3)	0.58
OSA	22.0 (-11.0–55.0)	0.018	30.0 (7.5–56.5)	< 0.001	9.0 (-9.5–31.0)	0.021

Serine						
Controls	5.2 (-0.3–13.4)	0.006	7.7 (-7.1–20.0)	0.016	1.5 (-13.0–14.4)	0.45
OSA	-7.0 (-18.4–2.6)	0.023	-13.1 (-20.0–0.0)	0.012	-3.0 (-14.3–11.3)	0.57
Threonine						
Controls	16.0 (1.0–31.3)	0.001	10.0 (-0.3–23.0)	0.008	-1.5 (-13.6–5.0)	0.18
OSA	-3.4 (-14.6–8.2)	0.23	-1.0 (-19.4–9.6)	0.47	3.0 (-11.2–12.0)	0.69
Tryptophan						
Controls	9.3 (6.4–16.1)	< 0.001	7.7 (4.9–15.6)	< 0.001	-0.6 (-7.5–3.5)	0.53
OSA	-0.1 (-9.6–13.0)	0.94	-1.8 (-9.7–11.0)	0.98	0.8 (-4.4–8.4)	0.96
Tyrosine						
Controls	9.4 (1.8–19.4)	< 0.001	12.7 (2.1–19.3)	< 0.001	-1.1 (-5.6–4.7)	0.96
OSA	-0.2 (-9.5–8.9)	0.74	1.2 (-9.2–11.2)	0.70	0.1 (-6.3–7.8)	0.91
Valine						
Controls	34.0 (18.0–54.8)	< 0.001	37.5 (8.3–53.3)	0.002	-1.5 (-14.3–17.0)	0.83
OSA	26.0 (-17.2–45.5)	0.17	-2.0 (-16.0–56.5)	0.19	8 (-8–19)	0.90
ADMA						

Controls	-0.03 (-0.1–0.06)	0.20	-0.04 (-0.14–0.03)	0.41	0.02 (-0.13–0.09)	0.86
OSA	-0.05 (-0.10–0.01)	0.002	-0.05 (-0.01–0.04)	0.019	0.01 (-0.07–0.08)	0.53
Creatinine						
Controls	4.2 (-0.7–8.3)	0.011	4.3 (-0.1–10.7)	0.012	-0.2 (-2.6–3.8)	0.67
OSA	2.7 (-3.5–8.0)	0.054	4.8 (-2.9–9.9)	0.052	2.5 (-3.0–5.4)	0.68
Kynurenine						
Controls	0.2 (-0.2–0.3)	0.24	-0.1 (-0.2–0.4)	0.52	-0.1 (-0.3–0.2)	0.68
OSA	-0.1 (-0.5–0.2)	0.17	-0.3 (-0.7–0.3)	0.042	-0.0 (-0.3–0.2)	0.26
Serotonin						
Controls	0.01 (0.0–0.15)	0.08	0.03 (0.0–0.1)	0.008	0.0 (-0.09–0.06)	0.44
OSA	0.02 (-0.02–0.07)	0.08	0.06 (-0.01–0.14)	0.12	0.0 (-0.06–0.1)	0.79
Taurine						
Controls	10.7 (-0.4–20.7)	0.001	9.4 (3.3–24.1)	< 0.001	1.6 (-8.7–17.7)	0.41
OSA	3.2 (-3.1–16.8)	0.07	12.7 (-0.5–25.3)	0.013	3.9 (-7.5–19.5)	0.16
Kyn/Trp ratio						
Controls	-0.005 (-0.009–-0.001)	< 0.001	-0.006 (-0.01–-0.003)	0.001	-0.0001 (-0.004–0.002)	0.75

OSA	-0.002 (-0.008–0.002)	0.11	-0.005 (-0.009–0.001)	0.01	-0.002 (-0.006–0.004)	0.23

*Statistical analysis has been performed with rank general linear model for repeated measures. Fisher's least significant difference (LSD) method was used for correction for multiple comparisons.

The model was adjusted to body mass index, age, gender, current smoking status, oxygen desaturation index including only desaturations with at least 5% drop in oxygen saturation; oxygen desaturation index including only desaturations extending below 90% and plasma contents of potassium, sodium, aspartate aminotransferase, alanine aminotransferase, urea, high-density lipoprotein cholesterol, low-density lipoprotein cholesterol and triglycerides.

ADMA asymmetric dimethylarginine, *hsCRP* high-sensitivity C-reactive protein.
